# Nasal Region Dimensions in Children: A CT Study and Clinical Implications

**DOI:** 10.1155/2014/125810

**Published:** 2014-05-05

**Authors:** Wirginia Likus, Grzegorz Bajor, Katarzyna Gruszczyńska, Jan Baron, Jarosław Markowski

**Affiliations:** ^1^Department of Human Anatomy, School of Medicine in Katowice, Medical University of Silesia, 18 Medyków Street, 40-752 Katowice, Poland; ^2^Department of Radiology and Nuclear Medicine, School of Medicine in Katowice, Medical University of Silesia, 18 Medyków Street, 40-752 Katowice, Poland; ^3^E.N.T. Department, School of Medicine in Katowice, Medical University of Silesia, 20-24 Francuska Street, 40-027 Katowice, Poland

## Abstract

Atresias of nasal cavity, especially in young children, pose an essential problem in children's otolaryngology. Only a few morphometric studies of nasal cavity concerning healthy neonates and young infants without nasal stenosis are available. Multislice computed tomography is a perfect tool enabling a precise evaluation of anatomic structures. The aim of this study was a complex morphometric evaluation of clinically important bone and mucosal structures of nasal cavity and examination of their dependence on age and sex in children up to 3 years of age. 180 children, age range 0–3 years, were divided into 5 age groups, and measurements of 18 distances between skeletal structures and between mucosal structures of nasal cavity were performed on their CT scans. A correlation between the widths of selected bone structures was examined. There were no statistically significant differences in analyzed morphometric parameters between adjacent age groups. The differences were statistically significant only between extreme age groups. There was a correlation between evaluated structures and age. Our results are a valuable supplement of nasal cavity morphometric data of young children. They may be useful in setting reference values of evaluated parameters in children and in diagnosis and planning of surgical treatment in children's otolaryngology.

## 1. Introduction

Breathing through the nose is the only physiological path for breathing in the first months of infant's life [1–5]. Thus, a slight stenosis in the nasal cavity may cause significant disturbances in the patency of the respiratory tract, which is a life-threatening condition in small children [6, 7]. Despite substantial clinical interest in the dimensions of the nasal cavity, in the aspect of stenoses [1, 6, 8, 9], as well as their vital practical importance, the number of anatomic studies, concerning nasal cavity dimensions in healthy children without problems with breathing through the nose, still remains insufficient. Computer tomography (CT) is presently the golden standard in diagnostics of nasal cavity diseases; thus, it can be applied successfully as a tool for assessing anatomical structures [10].

In case of problems with patency of the nose in children, the main pathology, which has to be excluded by using CT as tool, is choanal atresia (CA) [1, 11, 12]. Bilateral choanal atresia calls for immediate surgical intervention, as it poses a threat to the life of the newborn baby [1]. Computer tomography allows differentiating this disorder, juxtaposing it with disorders causing similar manifestations as* nasal obstruction without choanal atresia, *(NOWCA) [4, 7, 13].* Congenital nasal pyriform aperture stenosis* (CNPAS) is a rarely encountered bone-related stenosis occurring bilaterally and manifesting right after child birth [11, 13–16]. The CPNAS syndrome occurs rarely, yet it may coexist with many other developmental disorders [17]. It causes difficult breathing and may clinically mask posterior nares aplasia or stenosis. In order to diagnose it, it is indispensable to perform computer tomography examination [18–20]. Whereas diagnosing CA as a rule does not create difficulties, in case of diagnosing NOWCA it is necessary to find out whether the width of posterior nares in CT imaging is normal or diminished. That is why detailed knowledge of nasal cavity dimensions, both in its initial and final section, is indispensable from the clinical perspective of operative otorhinolaryngology. The decision concerning further treatment including surgical intervention is often based on the interpretation of CT images. Taking into account the fact that during the first three years of human life the tempo of body development is the fastest, thus it seems necessary to be able to compare the dimensions of measured structures with norms suitable for the age of examined children. Comparison of a wide range of morphometric dimensions between adjacent narrow age groups shall allow determining how wide the age range should be, for which the reference value is determined, so that it has possibly greatest clinical value, and in order not to create too narrow age groups, which would preclude their usefulness in diagnostics and clinical practice.

The measurements of clinically important distances in nasal cavity were performed in healthy children, some to several years ago, often on CT scans with layer thickness of 6-7 mm. Many of authors evaluated on CT scans dimensions of nasal cavity in wide age groups of neonates, infants, and even young children, which makes the data obtained that way often not very accurate, and which causes the necessity of verification. The layers of 0.5 mm in thickness, which we applied in CT examinations, make the results of our study more accurate, when compared with results obtained by other authors. The proper evaluation of nasal airways obstruction should be based on a comparison of patient's nasal cavity dimensions with precise “normal values.”

The aim of our study was to provide normal values and growth trends of the dimensions of various parts of nasal cavity, as well as to compare them in narrow age subgroups of children from 0 to 3 years of age without any nasal airways pathology. The knowledge of normal values of nasal cavity is necessary to understand the nature of nasal obstruction. Another aim of our study has been to determine how broad the age limits can be, for which the norms of examined anatomical structures should be established.

## 2. Materials and Methods

### 2.1. Patients

Retrospective studies have been performed on CT scans of 180 Polish Caucasian children (83 girls and 97 boys), with regular development of the brain, without any craniofacial abnormalities, the age range of whom was from birth to 3 years of age. All the patients were diagnosed between February 2009 and January 2012 in the Department of Medical Imaging, Radiology, and Nuclear Medicine, Medical University of Silesia, Katowice, Poland (Table 1).

Qualified for morphometric analyses were those children that were referred to head CT examination due to suspicion of head injury. Only those tomograms have been included in the assessment of nasal cavity dimensions, in which no deviations have been found from the standard condition of skull osseous structures. Excluded from the study have been the children born preterm, with alterations within the bones of skull, with genetic disorders (e.g., Down's syndrome), mental retardation, congenital defects and/or complexes of congenital defects (e.g., CHARGE association, VATER association, Apert syndrome, Crouzon syndrome, and Pfeiffer syndrome), craniosynostosis, and hydrocephalus.

The study group has been divided into 5 age categories: 0–3 months, 4–6 months, 7–12 months, under two years of age (13–24 months), and under three years of age (25–36 months) (Table 1).

An approval for this retrospective study was obtained from the Bioethics Commission of Medical University of Silesia.

*Nasal Region Dimensions*. The following 18 dimensions of the nasal cavity were measured to evaluate anterior, middle-third, and posterior widths and length of nasal fossae (Tables 2 and 3).

### 2.2. CT Protocol and Image Analysis

**Figure 1:**
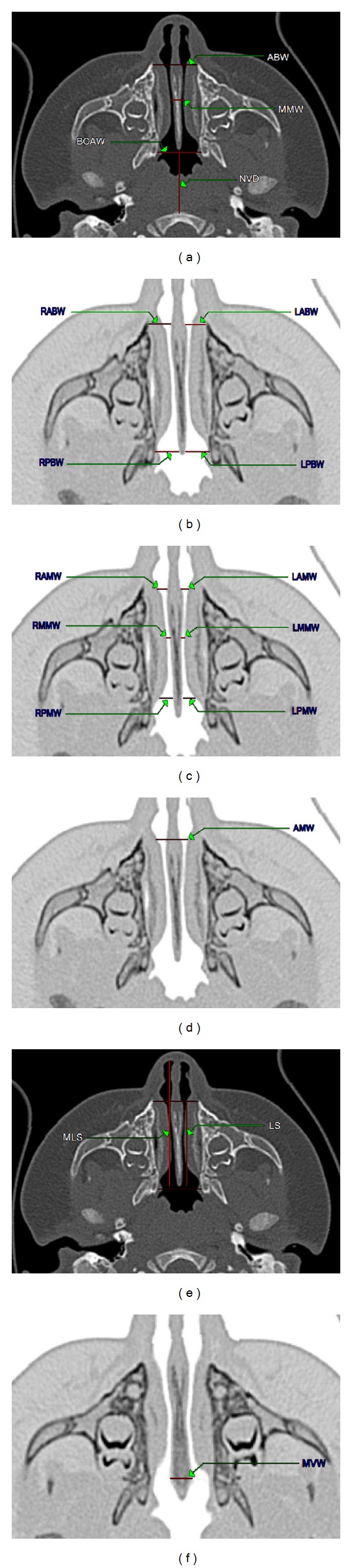
(a)–(f) Axial CT image normal nasal cavity. Measurements: (a) anterior bony width (ABW), minimal soft tissue (MMW), bony choanal aperture width (BCAW), and nasopharynx vertical distance (NVD); (b) right (RABW) and left (LABW) anterior bony width and right (RPBW) and left (LPBW) posterior bony width; (c) right (RAMW) and left (LAMW) anterior mucosal width and right (RMMW) and left (LMMW) minimal soft tissue width and right (RPMW) and left (LPMW) posterior mucosal width; (d) anterior mucosal width (AMW); (e) length of septum (LS) and maximal length of septum (MLS); (e) maximal width of vomer (MVW). Images (b), (c), (d), and (f) used color inversion.

CT examinations have been made using spiral technique, in transverse plane, in layers of 0.5 mm in thickness, using 64 row TOSHIBA Aquilion apparatus (Toshiba, Tokyo, Japan), in accordance with the standard diagnostic protocol for head examination. The obtained axial image from CT was transferred to a workstation for analysis. The measurement plane was parallel to the so-called Frankfurt plane (auriculo-orbital plane). Each scan was measured based on reliable bone and mucosa landmarks selected to assess the nasal region, as described by Aslan et al. and Contencin et al., and to obtain the measurements of clinically vital distances [1, 4]. The measurements were performed on scans at the level of nasal fossa floor and just above. In order to precisely visualize the air ducts, color inversion has been applied (Figures 1(b), 1(c), 1(d), and 1(f)).

All measurements were performed directly on CT films; each measurement was taken with the accuracy of 1/100 mm; measurements were standardized to a 5 cm reference scale on each film. The average of the two separate measurements was used for analysis.

### 2.3. Statistical Analysis

The compliance of the empirical distribution of the examined variables with normal distribution was assessed using the Shapiro-Wilk test. The homogeneity of variance was assessed using Levene's test. For the assessment of differences between the study age groups, as regards arithmetic mean, the ANOVA analysis of variance has been applied. For the assessment of significance of differences between the sexes in a given age group the Cochran Cox test has been applied. The statistical significance [of differences] between groups has been determined at the level of *P* ≤ 0.05.

## 3. Results

After application of strict exclusion criteria, 180 CT scans (83 female and 97 male ones) have been subjected to analysis (Table 1). Detailed results of all measurements (mean ± SD and 95% CI) have been listed in Tables 4, 5, 6, 7, 8, and 9.

**Figure 2:**
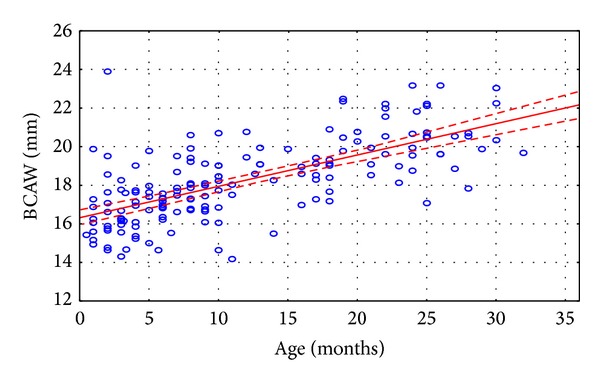
Graphs illustrating the width of bony choanal aperture (BCAW) of children without nasal cavity stenosis with 95% confidence intervals (*n* = 180).

The authors made the following observations:

- analyzing the correlation of parameters measured with the age of the child, a linear increase of the osseous parameters has been found, related to age, up to three years of age (Table 10). Only the biggest width of the vomer failed to increase under three years of age in the study group;
- for all the measured linear dimensions of bone and mucosa, no differences have been noted between the sexes, with the exception of the length of septum, which was bigger in case of girls in the age range of 0–3 months (Tables 4–9);
- analyzing the differences between the mean values of measured distances, for various age groups, the authors found that there were no statistically significant differences in analyzed morphometric parameters between adjacent age groups. The differences were statistically significant only between extreme age groups. There was a correlation between evaluated structures and age (Table 11);
- we noted increasing width of the posterior nares in children under the third year of age, which amounted to 0.162 mm/month (Figure 2).

Linear dependence between the child's age and the width of posterior nares can be found from the expression: choanal aperture [mm] = 16.317 + 0.16216 × age [months].

## 4. Discussion

The size of the nasal cavity is subject to personal differences; however, changes in size more often are due to pathological causes. Before starting treatment, mainly surgical treatment of nasal cavity stenoses, it is necessary to perform examinations employing imaging techniques, as well as to take morphometric measurements, in order to establish the degree of deviation from norm. Despite the substantial clinical significance of morphometric parameters of nasal cavity in children, the number of morphometric studies is still insufficient, studies which would assess the variability of nasal cavity dimensions with age, in newborns and small children, without difficulties with breathing through the nose.

Among the dimensions selected for the complex assessment of the nasal cavity, there were measurements having substantial clinical significance in diagnosing such disorders as CA, CNPAS, or NOWCA. Knowledge of the correct nasal cavity dimensions in children without problems with nose patency is indispensable, as studies conducted by various authors demonstrated changes in the size of nasal cavity structures in the disease entities described [1, 4, 19, 21, 22]. In the studies conducted by Belden et al. the pyriform aperture size as well as width of the nasal cavity was measured on CT images, in children under 12 months of age, with congenital nasal pyriform aperture stenosis [18]. It has been demonstrated that the width of the pyriform aperture, as well as the width of nasal cavity, was smaller, with statistical significance, in children with CNPAS in comparison with healthy children in the same age range [18]. CNPAS may be mistaken for posterior nares aplasia; thus, computer tomography is a useful diagnostic method, particularly for the assessment of stenosis degree. The studies described also confirmed reduction of posterior nares width in children with congenital nasal pyriform aperture stenosis. Lee et al. measured the width and height of the pyriform aperture in children under 4 months of age, on images obtained by three-dimensional computer tomography (3D-CT) [19]. They indicated the usefulness of CT imaging of the anterior part of nasal cavity, particularly in pre- and postoperative assessment in children. However, some authors point out that in the interpretation of nasal cavity dimensions, especially its width, the mucous membranes covering osseous structures ought to be taken into account. The presence of mucous membrane, of various widths, may contribute to changes in the diameter of the measured ducts [3, 19, 23–28].

CT allows differentiating between CA and NOWCA. In 1985, Slovis et al. established two parameters useful in the assessment of posterior nares stenosis (choanal atresia) in children [6]. They are the vomerine width and posterior nares width. According to the authors, vomerine width exceeding 0.67 mm would entail stenosis in that part of the nasal cavity in the child [6]. The particular significance of vomer width for the bilateral posterior nares atresia has also been stressed on in the research reported by Harnsberger as well as Vanzieleghem et al. [29, 30]. In the research conducted by Aslan et al. differences in the dimensions of nasal cavity structures have been determined, between children with bilateral posterior nares artesia and children without problems concerning nasal patency, under one year of age [1]. The authors quoted demonstrated that only the width of vomer and its surface area, as well as width of posterior nares, differed—with statistical significance—between the analyzed groups of children. In the study by Corsten et al., comprising 56 CT images obtained from children under one year of age, with and without problems with nasal patency, the differences in posterior nares width between analyzed groups of children have been demonstrated [8]. The measurements discussed, important not only for anthropometry, but also from the clinical point of view, have also been subject of analysis in our study. The analysis of results we obtained, as regards the nasal cavity dimensions measured at the level of the pyriform aperture, revealed positive correlation, of statistical significance, with the age of the child. The bone distance measured at the level of jaw nasal incisor in children between 6 and 12 months of age amounted to 17.110 ± 1.275 mm. For the same age group of children, the average distances measured from the jaw nasal incisor to the nasal septum mucosa on the right and left side amounted, respectively, to 7.047 ± 0.989 mm and 6.608 ± 0.898 mm. Lower values have been reported in the study of Aslan et al., who performed measurements on tomograms obtained from children under one year of age. The dimensions obtained for that part of the nasal cavity amounted in the study quoted, respectively, to 1.43 ± 0.16 cm and 0.58 ± 0.10 cm on the right side and 0.58 ± 0.13 cm on the left side [1]. Belden et al. assessed the dimensions of the pyriform aperture in children in three age groups: 0–3 months (13.4 mm), 4–6 months (14.9 mm), and 10–12 months (15.6 mm) [18]. Those authors demonstrated that dimensions increased with the age of the child. They obtained lower values of the parameters measured, in comparison with the results obtained for the same area of the nasal cavity in our study. For the age group between 0 and 3 months of age they amounted to 14.874 ± 1.620 mm. In the age group of 3 to 6 months, the result was 15.600 ± 1.57 mm, and in the age group between 6 and 12 months of age was 17.110 ± 1.275 mm. Contencin et al. analyzed 62 head CT images, obtained in children under 1 year of age [4]. The bone-to-bone dimensions of the pyriform aperture amounted to 13.24 ± 1.88 mm, and their value was also below the value obtained in our study, for the parameter described. Both Aslan et al. and Contencin et al. took measurements of distances between mucous membranes in the anterior part of the nasal cavity [1, 4]. The distance measured between lateral wall mucosa, corresponding to the AMW dimension in our study, amounted, in case of the authors quoted, to 8.73 mm and 0.94 ± 0.14 cm in children under one year of age, respectively. The analysis of linear parameters describing the distance between lateral wall mucosa and nasal septum mucosa on the right and left side demonstrated that they had a lower value, in comparison with the dimensions obtained in our study. They amounted to 8.54 mm for the AMW dimension, as well as 2.5 mm and 2.16 mm for RAMW and LAMW dimensions, respectively.

Analyzing the MMW dimension, which is the shortest distance between mucosa in the 1/3 of nasal cavity, we found that it did not differ—with statistical significance—between the age groups examined. Also in other studies, similar results have been obtained for that part of the nasal cavity [1]. For the RMMW and LMMW dimensions, we obtained higher values, in comparison with the values obtained by other authors [1, 4].

From clinical point of view, the posterior nares dimension is crucial. The values of posterior nares width on the right and left side (RPBW and LPBW), obtained in our research are close to the results obtained by Slovis et al. [6] amounting, respectively, to 0.67 cm in newborns, 0.70 cm in children under 2 years of age, and 0.75 cm in children under 4 years of age. Aslan et al. [1] obtained results similar to ours. Lang and Yilmaz et al. [31, 32] obtained smaller dimensions for healthy children. Assessing the bone-to-bone width of posterior nares, we obtained greater values of the morphometric parameter described (17.770 ± 1.452 mm), in comparison with the results obtained by other authors (e.g., 14.34 ± 1.8 mm [23]; 1.32 ± 0.14 cm) [1]. However, comparing the results obtained with results from other studies, it should be noted that measurements had been taken on selected heads [31] or the posterior nares had been measured in frontal plane, not in transverse plane [32].

A separate issue, which requires attention, is the measurements of the vomer. They are important in the aspect of posterior nares aplasia. There is no agreement among the authors, as to the optimum technique for taking vomer measurements. Depending upon the selected cutting plane, along which the vomer width is measured, that width can differ in the same patient. Also, there is no agreement as to the standard dimensions of vomer. Some authors assess the vomer width in transverse planes, at the level of hard palate [33]. In the study quoted, performed on a group of children under 8 years of age, the vomer width amounted to 2.8 mm. In the same study, the author states that it should not exceed 5.5 mm. The example of the study quoted proves how different the approach to vomer width assessment may be (2.8 mm–5.5 mm). Analyzing the available literature, one can notice that vomer dimensions taken 5 mm above the hard palate amount to, for example, 0.23 mm in the age group of 0–8 years of age and 0.28 mm in the age group of 8–20 years of age [6]. In our study, the biggest width of the vomer was measured. The vomer dimensions obtained in our study do not differ, with statistical significance, between the age groups. There is a substantial discrepancy between authors, as regards the norm for vomer dimensions. It may be less than 2.3 mm or less than 5.5 mm [29, 30]. Moosa et al. point out the technical difficulties related to vomer width measurements, related to partial ossification of cranial basis [33]. The anterior part of the facial skeleton undergoes complete ossification before the end of fourth year of life [34]. Our study was conducted on children under 3 years of age, when the ossification of all structures is not complete yet, which may be revealed in the discrepancies of results obtained [35]. The measurements results obtained in our study are higher than those reported in the studies quoted; however, those values are within the vomer size criteria proposed by Moosa et al. [33]. It should be pointed out that in our case the biggest width of vomer was measured.

The study conducted by Djupesland and Lyholm demonstrated that the size of the air space in the nasal cavity did not depend upon the sex, but had positive correlation—with statistical significance—with child head size [35]. The absence of statistically significant differences between the sexes, as regards the values of parameters measured, was also confirmed in our study. All the linear parameters of osseous structures in the nasal cavity measured in our study, with the exception of the vomer, revealed a positive correlation with the age of the child, with statistical significance. Our studies revealed that the air space in posterior nares changed under the age of three. In connection with the above, a linear dependence was determined, between the width of posterior nares and the age of child (Figure 2). The statistical analysis revealed that posterior nares width increased by 0.162 mm/week. The confirmation of the fact that posterior nares dimension increasing linearly with child's age can be found in the studies done by other authors [6, 8, 9]. Statistical analysis of the results of our measurements showed that in case of determining the norm for osseous parameters in the anterior and posterior part of the nasal cavity for the youngest children, the groups under 6 months of age may be joined in one age range and analyzed jointly. Similar results have been obtained for the eldest children studied (between 12 and 36 months of age). This may testify about uniform growth rate of the structures before 6 months of age, acceleration of growth from 6 to 12 months of age, and repeated deceleration of growth between 12 and 36 months of age (Table 11). The research work carried out so far, devoted to the measurement of morphometric parameters of nasal cavity, and determination of reference values for that area of the facial skeleton has been carried out on groups of children and youngsters, often comprising wide age ranges. They are often wider than the age ranges applied in our study: under 6 months of age [4], under one year of age [7], or under two years of age, as in the study by Belden et al. [18]. Such a manner of selecting the age group for the study, bearing in mind the size of the group, results in analyses being carried out often on small subgroups of children. That, in turn, drastically reduces the credibility of results obtained. As an example, we can provide here the study of Slovis et al. in which the width of posterior nares has been measured in only 5 newborns, while the width of vomer in 44 people, but in a very wide age range, from birth to the age of 8 years [6]. Such a study by the author quoted was for many years used as norm for posterior nares and vomer measurements, in the aspect of posterior nares stenosis [6].

A substantial percentage of authors refrains from analyzing results in smaller age groups, often joining them into one study group [1, 4, 9, 18, 32]. The above is due to the fact that the analysis of nasal cavity dimensions in case of persons without problems with breathing through the nose is performed due to the requirements of diagnostics of other diseases [1, 6, 18, 19]. Measurements of morphometric parameters of nasal cavity, for the purpose of this study, have been performed on CT head scans, prepared using a 64-row apparatus, in very thin layers, having the thickness of 0.5 mm. In the literature published so far, none of the reported measurements were performed on CT scans with such a tiny layer thickness. An additional advantage of the multislice computed tomography (MSCT), which was employed in our study, is the short examination time, which reduces the amount of artefacts related to movement, especially in the youngest patients.

Waitzman et al., gaining minimum differences (measuring error below 5%) in direct measurements of osseous structures on CT images of skulls, demonstrated the high level of credibility of measurements on CT images [36]. In connection with the above, measurements taken on CT scans allow for exact measurements of anatomic reference points, which may be successfully used, for example, by a surgeon, in planning surgical procedures and in reconstructions of osseous structures, for the purposes of maxillofacial surgery.

## 5. Conclusions

In summary, the complex results of measurements taken and presented in this report are of substantial importance for radiologists, in particular in the aspect of diagnosing stenoses within the cavity, or for simplifying the procedures to be performed by laryngologists in that anatomic area, including endoscopy or surgical procedures within the hard palate area in children (operative otolaryngology). The knowledge about the morphology of those structures is indispensable for interpretation of results of their imaging and provides valuable information for differential diagnostics. From the clinical perspective, it is also important to determine the reference values for the width of pyriform aperture, posterior nares, or vomer. Such information will surely prove useful for radiologists, who may have small patients suffering from choanal atresia, stenosis of the pyriform aperture, or other developmental anomalies within the facial skeleton, for which CT is the examination method of choice. The results we obtained allow us to delineate 3 age groups: 0–6 months, 7–12 months, and 13–36 months, for which different reference values of the anatomic structures assessed should be applied. The results presented by our team also constitute a new input to descriptive anatomy.

## Figures and Tables

**Table 1:** Study group.

Age group	Age categories	Female (*n*)	Male (*n*)	Total (*n*)
A	0–3 mo	13	10	23
B	4–6 mo	20	15	35
C	7–12 mo	27	26	53
D	2 years	14	28	42
E	3 years	9	18	27

	Total	83	97	180

**Table 2:** The nasal dimension measurements—anterior, middle-third and posterior part.

	Abbreviation	Measurement
Anterior nasal cavity	ABW	Anterior bony width between the two ridges extruding from the maxilla-pyriform aperture
	RABW*	Right anterior bony width from the right maxillary ridge to the septal mucosa
	LABW*	Left anterior bony width from the left maxillary ridge to the septal mucosa
	AMW*	Anterior mucosal width between two mucosal edges extruding from the maxilla including the anterior airspace and the global thickness of the septum
	RAMW*	Right anterior mucosal width between the lateral mucosa and the septal mucosa
	LAMW*	Left anterior mucosal width between the lateral mucosa and the septal mucosa

Middle-third nasal cavity	MMW	Minimal soft tissue width from the mucosa of one inferior turbinate to the other
	RMMW^∧^	Right minimal soft tissue width from the turbinal to the septal mucosa
	LMMW^∧^	Left minimal soft tissue width from the turbinal to the septal mucosa

Posterior nasal cavity	BCAW	Bony choanal aperture width between both pterygoid processes-choanal aperture
	RPBW^#^	Right posterior bony width between bone sidewall and septal mucosa
	LPBW^#^	Left posterior bony width between bone sidewall and septal mucosa
	RPMW^#^	Right posterior mucosal width between the lateral mucosa and the septal mucosa
	LPMW^#^	Left posterior mucosal width between the lateral mucosa and the septal mucosa
	MVW	Maximal width of vomer

*Measurements on the same line to ABW distance; ^∧^measurements on the same line to MMW distance. ^#^Measurements on the same line to BCAW distance.

**Table 3:** The nasal dimension measurements and nosopharynx.

	Abbreviation	Measurement
Nasal septum	LS	Length of septum between pyriform aperture to the end of the vomer
	MLS	Maximal length of septum between the most anterior part of nasal septum to the end of the vomer

Nasopharynx	NVD	Nasopharynx vertical distance between posterior vomer and cranial base

**Table 4:** Anterior nasal cavity dimensions—interosseuos distances.

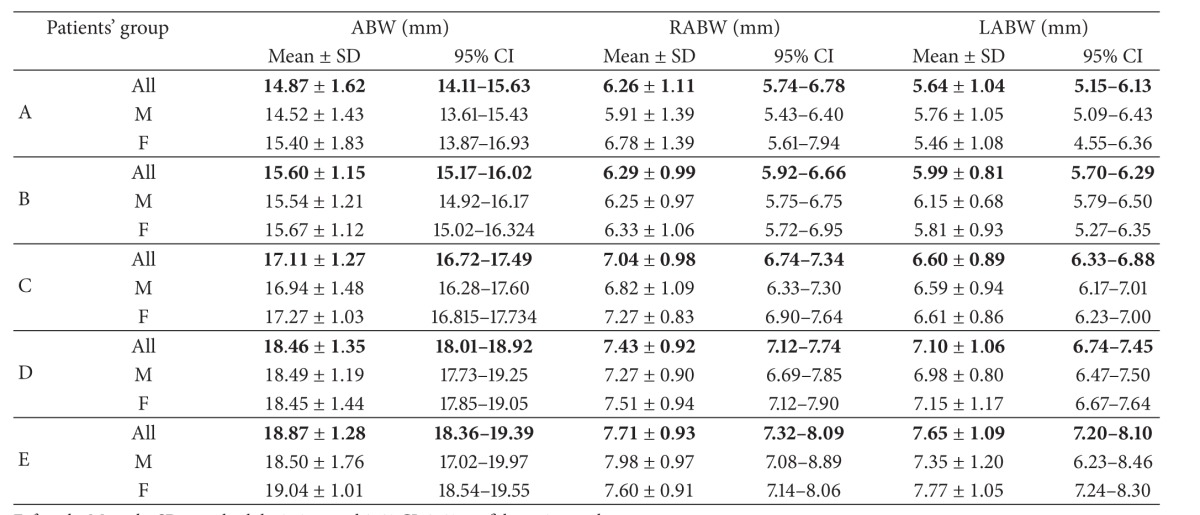

**Table 5:** Anterior part of the nasal cavity—intermucous distances.

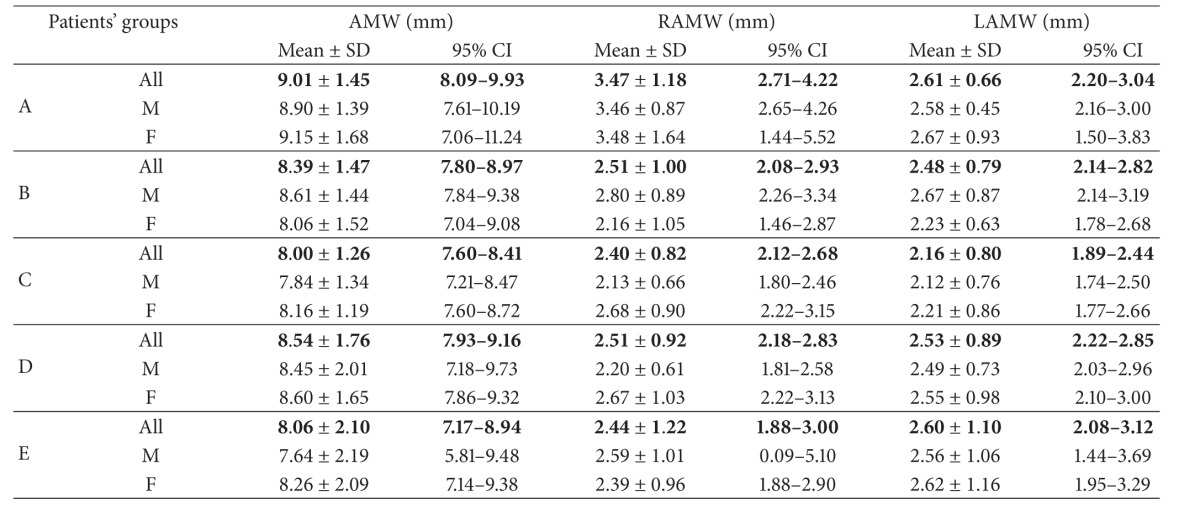

**Table 6:** Central 1/3 part of the nasal cavity.

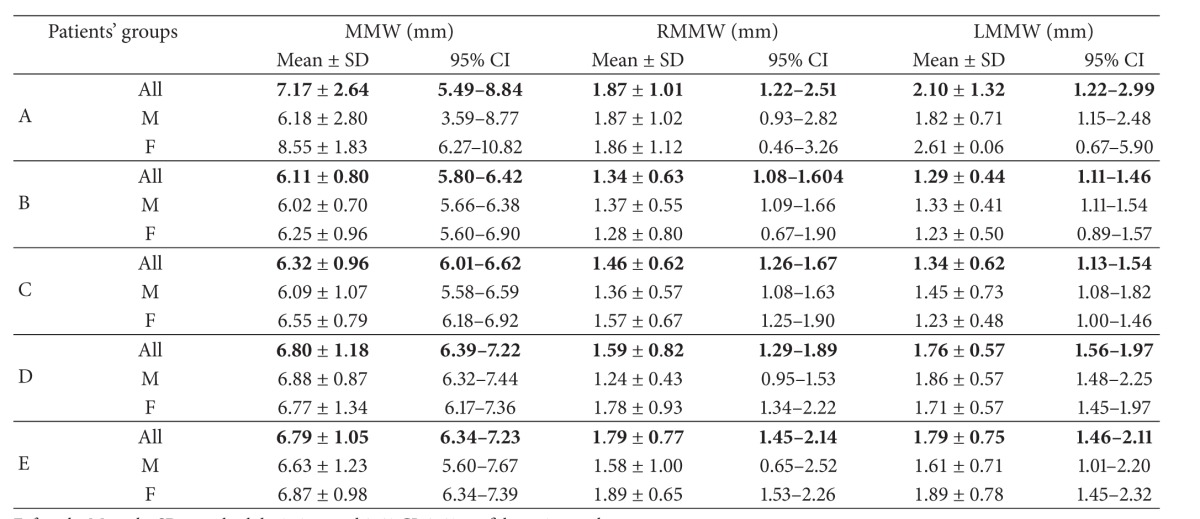

**Table 7:** Posterior part of the nasal cavity—interosseous distances.

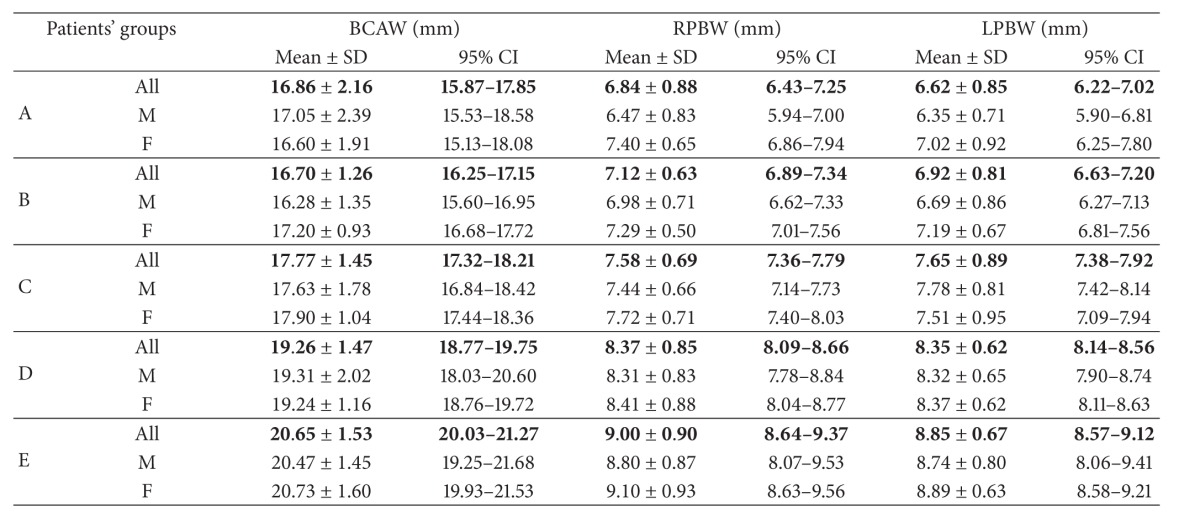

**Table 8:** Posterior part of nasal cavity—intermucous distances.

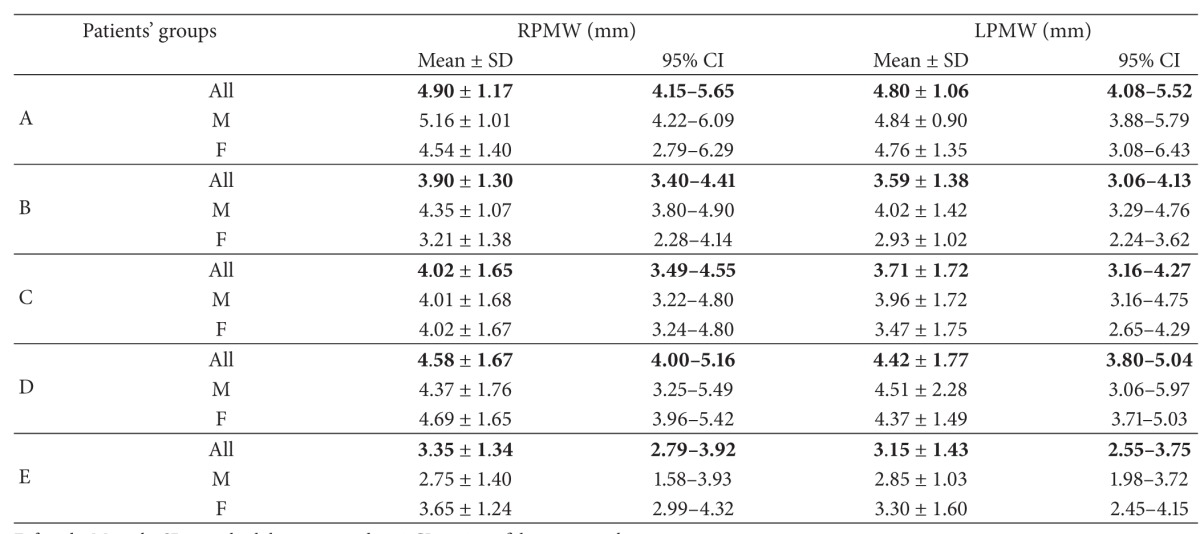

**Table 9:** Nasal septum and vomer.

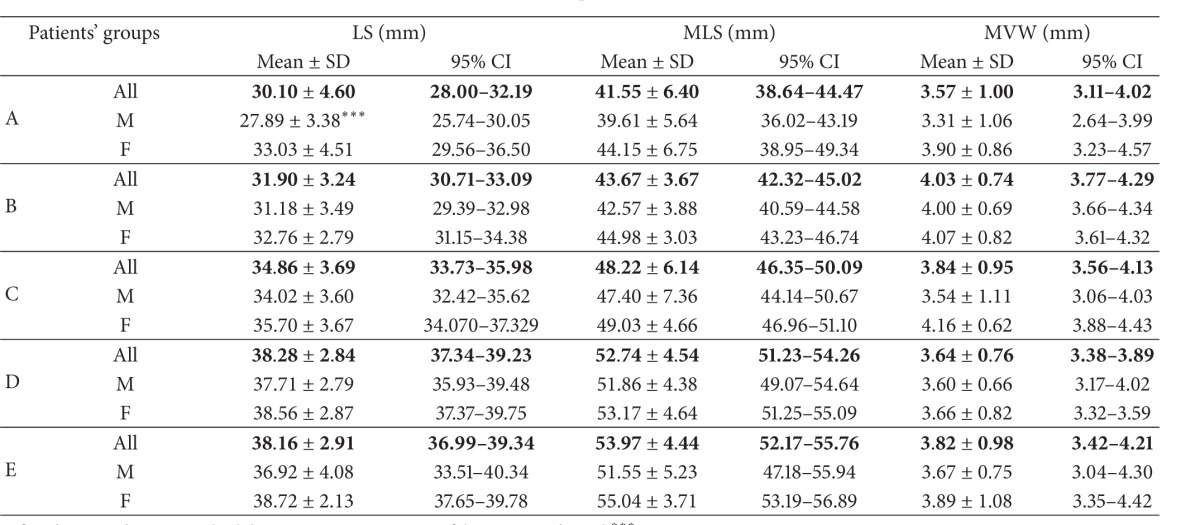

**Table 10:** Value of the Pearson linear correlation coefficient (*r*_*xy*_) for nasal cavity versus age of the child, *P* < 0.05.

Parameter	*r*_*xy*_
ABW	0.734
LPBW	0.701
MLS	0.691
BCAW	0.687
LS	0.669
RPBW	0.661
LABW	0.564
RABW	0.475
MSW	0.471
NVD	0.351
MVW	No correlation

**Table 11:** Statistical significance of differences between average values for the measured linear parameters of the nasal cavity (*P*).

ABW	B	C	D	E	LPBW	B	C	D	E
A	ns	∗∗∗	∗∗∗	∗∗∗	A	ns	∗∗∗	∗∗∗	∗∗∗
B		∗∗∗	∗∗∗	∗∗∗	B		∗∗∗	∗∗∗	∗∗∗
C			∗∗∗	∗∗∗	C			∗∗∗	∗∗∗
D				ns	D				ns

RABW	B	C	D	E	RPMW	B	C	D	E

A	ns	ns	∗∗∗	∗∗∗	A	ns	ns	ns	ns
B		∗∗∗	∗∗∗	∗∗∗	B		ns	ns	ns
C			ns	ns	C			ns	ns
D				ns	D				∗

LABW	B	C	D	E	LPMW	B	C	D	E

A	ns	∗∗	∗∗∗	∗∗∗	A	ns	ns	ns	ns
B		ns	∗∗∗	∗∗∗	B		ns	ns	ns
C			ns	∗∗	C			ns	ns
D				ns	D				∗

LMMW	B	C	D	E	LS	B	C	D	E

A	∗	ns	ns	ns	A	ns	∗∗∗	∗∗∗	∗∗∗
B		ns	ns	ns	B		∗∗∗	∗∗∗	∗∗∗
C			ns	ns	C			∗∗∗	∗∗∗
D				ns	D				ns

BCAW	B	C	D	E	MLS	B	C	D	E

A	ns	ns	∗∗∗	∗∗∗	A	ns	∗∗∗	∗∗∗	∗∗∗
B		∗	∗∗∗	∗∗∗	B		∗∗∗	∗∗∗	∗∗∗
C			∗∗∗	∗∗∗	C			∗∗∗	∗∗∗
D				∗	D				ns

RPBW	B	C	D	E					

A	ns	∗	∗∗∗	∗∗∗					
B		ns	∗∗∗	∗∗∗					
C			∗∗∗	∗∗∗					
D				∗					

A–E: patients age groups, *P ≤ 0.05; **P < 0.01, ***P < 0.001, and ns: not significant.
